# Arbuscular mycorrhizal fungi and phosphorus supply accelerate main medicinal component production of *Polygonum cuspidatum*

**DOI:** 10.3389/fmicb.2022.1006140

**Published:** 2022-09-08

**Authors:** Rui-Ting Sun, Ze-Zhi Zhang, Ming-Yang Liu, Xiang-Cao Feng, Nong Zhou, Hai-Dong Feng, Abeer Hashem, Elsayed Fathi Abd_Allah, Wiwiek Harsonowati, Qiang-Sheng Wu

**Affiliations:** ^1^College of Horticulture and Gardening, Yangtze University, Jingzhou, China; ^2^Shiyan Academy of Agricultural Sciences, Shiyan, China; ^3^College of Biology and Food Engineering, Chongqing Three Gorges University, Chongqing, China; ^4^Department of Botany and Microbiology, College of Science, King Saud University, Riyadh, Saudi Arabia; ^5^Department of Plant Production, College of Food and Agricultural Sciences, King Saud University, Riyadh, Saudi Arabia; ^6^Department of Agrobiology and Bioresources, School of Agriculture, Utsunomiya University, Utsunomiya, Tochigi, Japan

**Keywords:** arbuscular mycorrhiza, medicinal plant, metabolite, P stress, resveratrol

## Abstract

The medicinal plant *Polygonum cuspidatum* Sieb. Et Zucc is rich in stilbenes (e.g., polygonin and resveratrol) and anthraquinones (e.g., emodin) for the therapy of human diseases, while how to increase the growth and medicinal composition concentrations of *P*. *cuspidatum* has become an urgent issue. The aim of the present study was to evaluate the effects of inoculation with an arbuscular mycorrhizal (AM) fungus, *Funneliformis mosseae*, on plant growth, phosphorus (P) acquisition, medicinal component concentrations, and expressions of resveratrol synthesis-associated enzyme genes of *P*. *cuspidatum* at two P levels (0 M and 0.2 M). P supply (0.2 M) stimulated root AM fungal colonization rate. *F*. *mosseae* inoculation significantly improved growth performance (height, diameter, and biomass) and root morphology (diameter, length, and projected area), irrespectively of substrate P levels. P supply and *F*. *mosseae* distinctly increased soil acid and neutral phosphatase activities, as well as root P concentrations. P supply increased root physcion and resveratrol concentrations in inoculated and uninoculated plants, along with up-regulated expressions of *PcCHS1*, *PcCRS1*, *PcRS11*, and *PcSTS*. AM plants represented significantly higher root aloe-emodin, chrysophanol, emodin, physcion, polydatin, and resveratrol concentrations than non-AM plants irrespective of P levels, coupled with up-regulated expressions of *PcCHS1*, *PcCHS2*, *PcRS11*, *PcRS*, and *PcSTS*. It is concluded that 0.2 M P supply and *F*. *mosseae* inoculation promoted chrysophanol, physcion, polydatin, and resveratrol concentrations of *P*. *cuspidatum*, with the increase in resveratrol associated with up-regulated expressions of related genes.

## Introduction

*Polygonum cuspidatum* Sieb. Et Zucc is a medicinal plant, whose active ingredients contain stilbene compounds (e.g., resveratrol) and anthraquinones (e.g., emodin) (Kong et al., 2020). In East Asia, *P*. *cuspidatum* is widely used in the therapy of hepatitis, cough, jaundice, and other diseases (Zhang et al., 2013). Resveratrol, emodin, physcion, and chrysophanol of *P*. *cuspidatum* have been used as monomeric ingredients in pharmaceutical, chemical, and food applications, and thus *P*. *cuspidatum* becomes the raw material for these monomeric ingredients (Sun, 2007). Among them, resveratrol can not only improve the disease resistance of plants, but also have antioxidant and anti-tumor effects, along with great demand market (Wu et al., 2019); emodin has the functions of lowering blood pressure, protecting liver and anti-tumor, and immunomodulatory effects (Wu et al., 2022). Of all reported plant species, *P*. *cuspidatum* has much greater resveratrol concentrations than other plants (Wu et al., 2019).

Arbuscular mycorrhizal (AM) fungi are beneficial fungi in soil that colonize the roots of approximately 72% of terrestrial plants, thus establishing a reciprocal symbiosis (Genre et al., 2020). AM fungi affect primary metabolic processes of plants and also change secondary metabolites in various medicinal plants (Zeng et al., 2014; Sun et al., 2021). In *Glycyrrhiza uralensis* plants, AM fungi distinctly raised concentrations of glycyrrhizic acid, and the increase became greater with the prolongation of inoculated time (Liu et al., 2007). In *Angelica dahurica* plants, AM fungi also dramatically induced the increase in total coumarin and imperatorin concentrations (Zhao and He, 2011). However, in *Ocimum basilicum* plants, *Glomus intraradices* triggered an increase in anthocyanin levels, but it did not affect concentrations of polyphenolic substances (Lee and Scagel, 2009). These results imply the potential of AM fungi in increasing specific medicinal ingredients in certain medicinal plants.

Substrate phosphorus (P) levels have an impact on the colonization response of AM fungi (Campos et al., 2018; Cao et al., 2021). High levels of substrate P inhibit AM fungal colonization (Breuillin et al., 2010), and low levels of P and AM fungi have improved effects on the accumulation of secondary metabolites in plants (Zeng et al., 2014). Inoculation with *G*. *mosseae* promoted artemisinin content in the medicinal plant *Artemisia annua* under low P conditions (40 mg/kg), while high P (120 mg/kg) significantly decreased artemisinin content by 42.5% (Tan et al., 2013). P fertilizer supply could achieve similar results to AM fungal inoculation in promoting the content of active ingredients in medicinal plants, such as castanospermine levels in *Castanospermum austral* plants and essential oils in *Foeniculum vulgare* plants (Kapoor et al., 2004; Bastami and Majidian, 2016). In *Anadenanthera colubrina* seedlings, AM fungi had an increased effect on the content of total phenols, total flavonoids, and total tannins in leaves, even under high P supply (30 and 50 mg/dm^3^) (Pedone-Bonfim et al., 2013). Interestingly, *G*. *mosseae* inoculation, but not P fertilization application, accelerated the production of essential oils in oregano plants (Khaosaad et al., 2006), suggesting different potential mechanisms for P fertilization and AM fungi to affect secondary metabolism.

*Polygonum* *cuspidatum* is mostly planted in mountainous areas, where the soil is poor, especially with low levels of P (Yu, 2016). An AM fungus, *Funneliformis mosseae*, could establish mycorrhizal symbionts in roots of *P*. *cuspidatum* and improve plant growth and root development, coupled with the increase in root medicinal components (Sun et al., 2022a). However, it is not clear whether the AM fungus promotes these medicinal components of *P*. *cuspidatum* under different P levels and what the underlying molecular mechanisms are involved in the promotion of medicinal components. The purpose of the present study was to assess the effects of *F*. *mosseae* on plant growth, P acquisition, soil phosphatase activity, concentrations of medicinal ingredients, and relative expressions of related genes in potted *P*. *cuspidatum* under low and appropriate P conditions.

## Materials and methods

### Plant culture and experimental design

Seeds of *P*. *cuspidatum* (identification: WUK 0310891)^1^ (provided by the Shiyan Academy of Agricultural Sciences) were disinfected in 75% alcohol solutions for 8 min and sown in 2.4 l plastic pots supplied with 4 mm sieved autoclaved (0.11 Mpa, 2 h) soil. After 4 weeks, seedlings with four leaves were selected for transplanting and were also inoculated with an AM fungus at the same time. The AM fungal strain used was *F*. *mosseae* (BGC XZ02A), whose origin and propagation were reported by Sun et al. (2022a). The plastic pot for transplanting had the size of 21 cm × 18 cm × 12 cm, together with 2050 g of pre-acid-eluted autoclaved river (Φ < 4 mm) sand. AM fungal inoculation received 150 g of AM fungal inoculum (2,850 spores), while non-inoculation with AM fungus also supplied 150 g of autoclaved mycorrhizal inoculum plus 2 ml 30 μm filtrates of the inoculum, aiming to maintain consistent microbes except for this AM fungus. After transplanting, the treated seedlings were allowed to grow indoors for 4 days and then transferred to a controlled greenhouse for growth, whose environmental conditions have been described in detail by Sun et al. (2022a).

P treatments were carried out after 3 weeks. P levels of 0.2 M (pH 6.5) and 0 M were selected by varying the level of P in the Hoagland nutrient solution (Hoagland, 1950) with 70 ml/pot. P supply was performed at 2-day intervals, and a total of 17 supplies of P were made. This experiment was conducted in a completely randomized block design with inoculation and non-inoculation of *F*. *mosseae* and two P levels, including low (0 M; P_0_) and appropriate (0.2 M; P_0.2_) P treatments (Song et al., 2006). As a result, the experiment consisted of four treatments, each of which was replicated eight times, with a total of 32 pots. The experiment ended after 12 weeks.

### Determinations of plant growth and root morphology

Plant growth traits (height, diameter, and leaf number) were determined directly before harvesting. After harvesting, plants were split into shoots and roots and then weighed individually. The complete root system was scanned using a scanner (J221A, EPSON, Jakarta Selatan, Indonesia), and the root images obtained were used to analyze root morphological parameters as per a WinRHIZO software (Regent Instruments Inc., Quebec, Canada), including length, area, volume, and diameter.

### Determinations of root AM fungal colonization rate

After roots were scanned, fresh root segments were cleared by 10% potassium hydroxide solution at 95°C for 2 h, bleached with 30% hydrogen peroxide solution for 15 min, and stained with 0.05% trypan blue in lactic acid for 30 s for microscopic observation (Phillips and Hayman, 1970). The estimation of the AM fungal colonization rate was performed using the protocol outlined by Zou et al. (2021).

### Determinations of root p concentration and soil phosphatase activity

The content of P in roots was determined using the colorimetric method outlined by Cavell (1955), after dried root samples were digested with H_2_SO_4_-H_2_O_2_. Soil phosphatase activity was assayed using the procedure outlined by Wu et al. (2015a), in which acid, neutral, and alkaline phosphatases were extracted with acetate buffer (pH 5.0), citrate–phosphate buffer (pH 7.0), and borate buffer (pH 10.0), respectively.

### Determinations of root medicinal ingredients

The extraction of root medicinal ingredients (aloe-emodin, chrysophanol, emodin, physcion, polydatin, and resveratrol) was performed by sonicating 10 ml of 80% methanol with 0.20 of dried root samples (Φ < 4 mm) for 30 min, followed by centrifugation at 4000 ×*g* for 10 min. The supernatant was filtered through a 0.22 μm filter membrane and assayed by high-performance liquid chromatography (HPLC) (LC-20A, Shimadzu, Japan). The chromatographic condition was performed according to the protocol reported by Sun et al. (2022a).

### Determinations of gene expressions

Total RNA in roots was extracted using a Quick RNA Isolation Kit (Huayueyang), referring to the user manual. The RNA was reversely transcribed into cDNA using the TRUE 1st Strand cDNA Synthesis Kit with gDNA Eraser kit (Aidlab). The six genes associated with the synthesis of medicinal ingredients were screened in the NCBI database, and then Primer Premier 5.0 was used to design primer sequences (Supplementary Table S1) for selected genes. qRT-PCR was conducted according to the protocol of Sun et al. (2022b). Each gene had three biological replicates, and each biological replicate contained three technical replicates. The relative expression of genes was calculated by the 2^-ΔΔCt^ method (Livak and Schmittgen, 2001), normalized to the treatment with non-AM fungal colonization under P_0_ levels.

### Statistical analysis

Before data analysis, homogeneity of variance was performed according to Levine’s test. Subsequently, two-way analysis of variance was performed on the experimental data using the SAS® software, and Duncan’s Multiple Range test was performed to compare significant differences (*p* < 0.05). Figures were made using SigmaPlot software and Adobe Photoshop CS6.

## Results

### Effects of P supply on root AM fungal colonization rate

**Figure 1:**
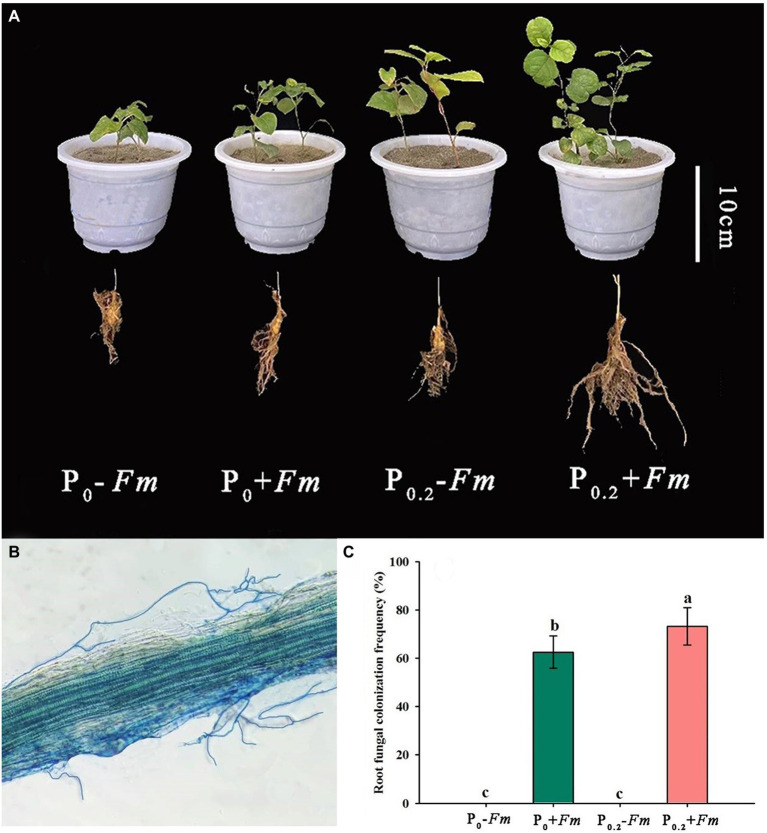
Plant growth responses **(A)**, root fungal colonization **(B),** and changes in root fungal colonization rate **(C)** of *Polygonum cuspidatum* to *Funneliformis mosseae* and P supply. Data (means ± SD, *n* = 4)followed by different letters above the bars indicate significant (*p* < 0.05) differences among treatments. +*Fm*, inoculation with *F. mosseae*; −*Fm*, inoculation without *F*. *mosseae*; P_0_, 0 M P; P_0.2_, 0.2 M P; P, phosphorus.

AM fungal colonization was found in *F*. *mosseae*-inoculated roots of *P*. *cuspidatum* (Figure 1B), regardless of substrate P levels, accompanied by 62.5–73.2% of root AM fungal colonization rate. Among them, P supply significantly increased root AM fungal colonization rate by 17.1% (Figure 1C). No significant interaction between AM fungal inoculation and P supply was observed in root AM fungal colonization (Table 1).

**Table 1:** Significance of variables between *Funneliformis mosseae* and P supply.

Variables	P supply	*Fm* inoculation	P supply × *Fm* inoculation	Variables	P supply	*Fm* inoculation	P supply × *Fm* inoculation
Root fungal colonization	0.0592	<0.0001	0.0592	Polydatin	<0.0001	<0.0001	<0.0001
P levels	<0.0001	0.0006	0.4948	Resveratrol	0.0014	<0.0001	0.0101
Soil acid phosphatase	0.0126	0.0003	0.5313	*PcCHS1*	<0.0001	<0.0001	0.8534
Soil neutral phosphatase	0.0016	<0.0001	0.4323	*PcCHS2*	0.0680	<0.0001	0.0008
Soil alkaline phosphatase	0.0716	0.0026	0.5182	*PcCRS1*	<0.0001	<0.0001	0.0002
Aloe-emodin	0.9348	<0.0001	0.0032	*PcRS11*	<0.0001	0.0008	0.7752
Chrysophanol	0.0140	0.0002	0.1471	*PcRS*	<0.0001	<0.0001	0.0009
Emodin	0.2796	<0.0001	0.0101	*PcSTS*	<0.0001	<0.0001	<0.0001
Physcion	0.7045	0.0021	<0.0001				

*Fm*, *Funneliformis mosseae*; P, phosphorus; CHS, chalcone synthase; RS, resveratrol synthase; STS, stilbene synthase; CRS, supposed stilbene synthase; RS11, resveratrol-forming stilbene synthase 11.

### Effects of AM fungi and P supply on plant growth performance

P supply and AM fungal inoculation significantly improved plant growth performance (Figure 1A; Table 2). Under P_0_ conditions, *F*. *mosseae* significantly increased height, stem diameter, number of leaves, shoot biomass, and root biomass by 18.5, 17.0, 31.8, 42.6, and 11.6%, respectively; under P_0.2_ conditions, *F*. *mosseae* significantly increased height, stem diameter, shoot biomass and root biomass by 18.8, 24.2, 45.7 and 93.8%, respectively (Table 2). A significant interaction was observed in root biomass. The combination of P supply and *F*. *mosseae* inoculation represented greater plant growth traits than other treatments.

**Table 2:** Effects of *Funneliformis mosseae* and P supply on plant growth performance of *Polygonum cuspidatum* Sieb. et Zucc.

Treatments	Height (cm)	Stem diameter (mm)	Leaf number (num./plant)	Biomass (g/plant)	
				Shoot	Root
P_0_-*Fm*	9.24 ± 0.98c	1.77 ± 0.19c	4.4 ± 0.5c	1.08 ± 0.09c	1.89 ± 0.25c
P_0+_*Fm*	10.95 ± 1.28b	2.07 ± 0.22b	5.8 ± 0.8b	1.54 ± 0.16b	2.11 ± 0.28b
P_0.2_-*Fm*	11.50 ± 1.34b	1.98 ± 0.19bc	8.6 ± 0.9a	1.38 ± 0.16b	2.59 ± 0.31b
P_0.2+_*Fm*	13.66 ± 1.16a	2.46 ± 0.23a	8.8 ± 0.8a	2.01 ± 0.25a	5.02 ± 0.55a
*Significance*					
P supply	0.0023	0.0006	0.0379	0.0007	<0.0001
*Fm* inoculation	0.0003	0.0046	<0.0001	<0.0001	<0.0001
P supply × *Fm* inoculation	0.6793	0.3404	0.1090	0.3473	<0.0001

Data (means ± SD, *n* = 4) followed by different letters in the column indicate significant differences (*p* < 0.05). *+Fm*, inoculation with *Funneliformis mosseae*; *−Fm*, inoculation without *F. mosseae*; P_0_, 0 M P; P_0.2_, 0.2 M P; P, phosphorus.

### Effects of AM fungi and P supply on root morphology

P supply and AM fungal inoculation significantly promoted root morphological architecture, and P supply into AM plants exhibited greater positive effects than non-AM plants (Table 3). *F*. *mosseae* significantly increased root average diameter, total length, and projected area by 18.1, 22.3, and 11.2% under P_0_ conditions and 26.4, 84.4, and 62.8% under P_0.2_ conditions. However, *F*. *mosseae* did not significantly improve root surface area and volume, independent of substrate P levels. There were significant interactions between AM fungal inoculation and P supply in root total length and projected area.

**Table 3:** Effects of *Funneliformis mosseae* and P supply on root morphology of *Polygonum cuspidatum*.

Treatments	Average diameter (mm)	Total length (cm)	Projected area (cm^2^)	Surface area (cm^2^)	Volume (cm^3^)
P_0_-*Fm*	2.27 ± 0.24c	62.17 ± 6.05c	5.69 ± 0.52c	10.16 ± 1.26b	0.82 ± 0.09c
P_0_ + *Fm*	2.68 ± 0.27b	76.01 ± 7.87b	6.33 ± 0.65b	11.77 ± 1.14ab	1.23 ± 0.13bc
P_0.2_-*Fm*	2.54 ± 0.27bc	71.43 ± 7.28bc	5.96 ± 0.64bc	12.81 ± 1.51a	2.68 ± 0.27a
P_0.2_ + *Fm*	3.21 ± 0.33a	131.7 ± 14.83a	9.70 ± 1.31a	13.30 ± 1.49a	2.94 ± 0.31a
*Significance*					
P supply	0.0055	<0.0001	0.0002	0.0034	<0.0001
*Fm* inoculation	0.0005	<0.0001	<0.0001	0.1022	0.0042
P supply × *Fm* inoculation	0.3009	<0.0001	0.0008	0.3713	0.4460

Data (means ± SD, *n* = 4) followed by different letters in the column indicate significant differences (*p* < 0.05). *+Fm*, inoculation with *Funneliformis mosseae*; *−Fm*, inoculation without *F. mosseae*; P_0_, 0 M P; P_0.2_, 0.2 M P; P, phosphorus.

### Effects of AM fungi and P supply on root P levels and soil phosphatase activities

**Figure 2:**
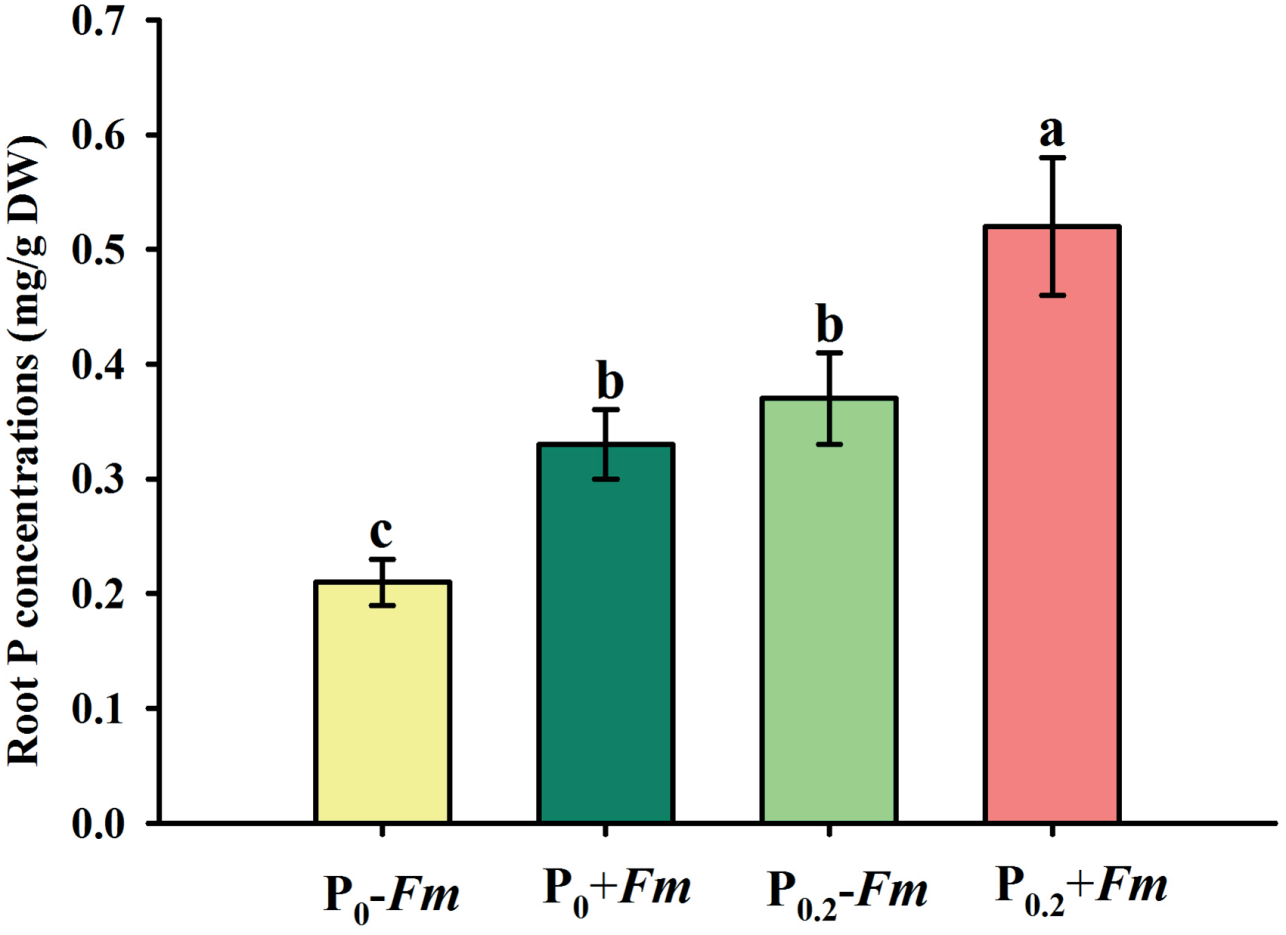
Changes in root P concentration of *Polygonum cuspidatum* by *Funneliformis mosseae* and P supply. Data (means ± SD, *n* = 4) followed by different letters above the bars indicate significant (*p* < 0.05) differences among treatments. +*Fm*, inoculation with *F. mosseae*; −*Fm*, inoculation without *F*. *mosseae*; P_0_, 0 M P; P_0.2_, 0.2 M P; P, phosphorus.

**Figure 3:**
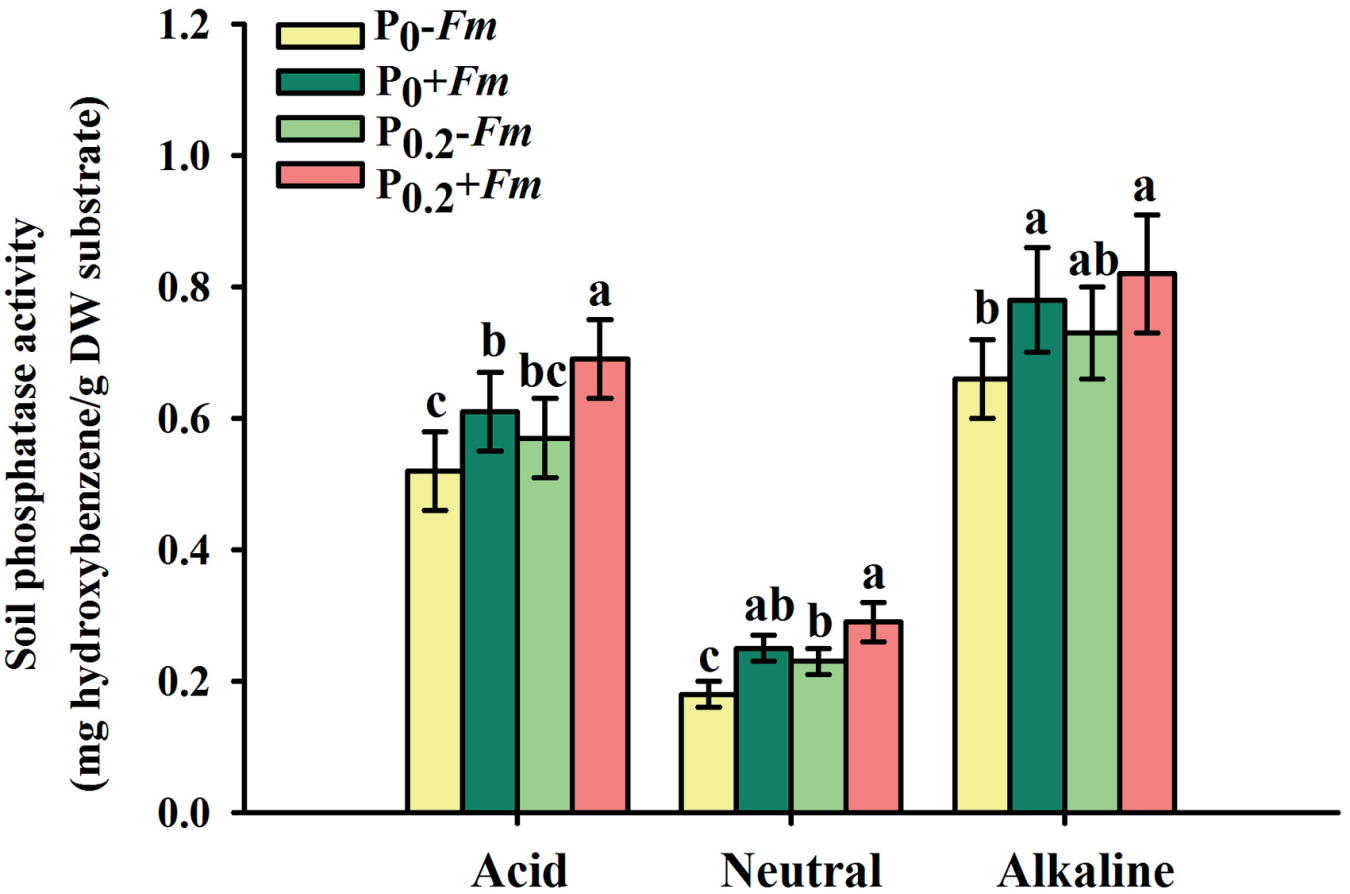
Changes in soil phosphatase activities of *Polygonum cuspidatum* by *Funneliformis mosseae* and P supply. Data (means ± SD, *n* = 4) followed by different letters above the bars indicate significant (*p* < 0.05) differences among treatments. +*Fm*, inoculation with *F. mosseae*; −*Fm*, inoculation without *F*. *mosseae*; P_0_, 0 M P; P_0.2_, 0.2 M P; P, phosphorus.

P supply dramatically increased root P levels, irrespective of AM fungal inoculation or not (Figure 2). *Funneliformis* *mosseae* inoculation significantly raised root P levels by 57.1 and 40.5% under P_0_ and P_0.2_ levels conditions, respectively. P supply and AM fungal inoculation, to a certain extent, improved soil acid, neutral and alkaline phosphatase activities (Figure 3). Under P_0_ conditions, *F*. *mosseae* significantly increased soil acid, neutral and alkaline phosphatase activities by 17.3, 38.9, and 18.2%, respectively; under P_0.2_ conditions, *F*. *mosseae* significantly elevated soil acid and neutral phosphatase activities by 21.1 and 26.1%, respectively. There was a significant interaction in root total length and projected area (Table 1). It indicated that dual application of P supply and *F*. *mosseae* exhibited greater increased magnitude that single application.

### Effects of AM fungi and P supply on concentrations of root medicinal ingredients

**Figure 4:**
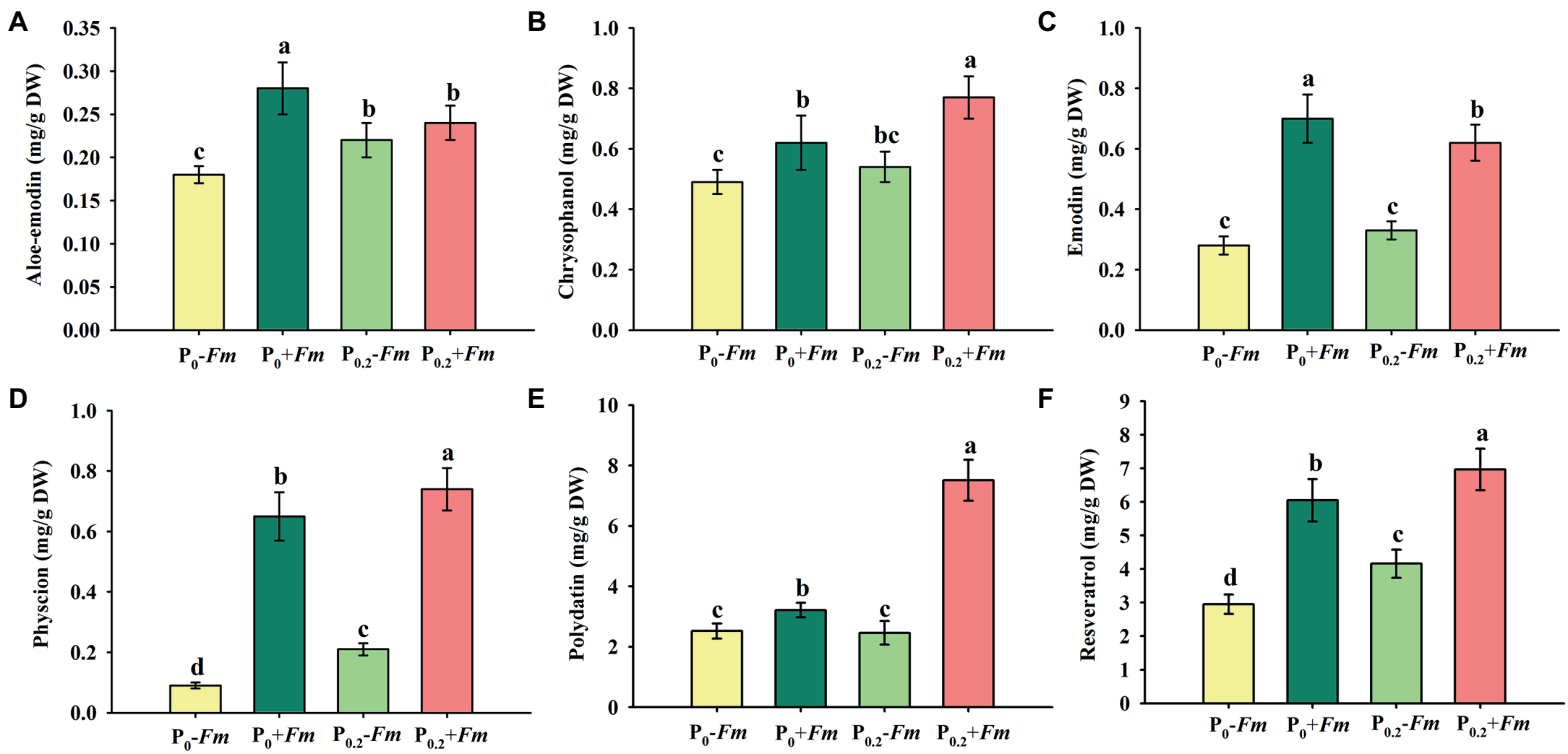
Changes in root aloe-emodin **(A)**, chrysophanol **(B)**, emodin **(C)**, physcion **(D)**, polydatin **(E)**, and resveratrol **(F)** of *Polygonum cuspidatum* by *Funneliformis mosseae* and P supply. Data (means ± SD, *n* = 4) followed by different letters above the bars indicate significant (*p* < 0.05) differences among treatments. +*Fm*, inoculation with *F. mosseae*; −*Fm*, inoculation without *F*. *mosseae*; P_0_, 0 M P; P_0.2_, 0.2 M P; P, phosphorus.

P supply dramatically increased root chrysophanol, physcion, polydatin, and resveratrol concentrations in AM plants, along with the increase in aloe-emodin, physcion, and resveratrol concentrations in non-AM plants (Figures 4A–F). Under P_0_ conditions, *F*. *mosseae* inoculation significantly raised root aloe-emodin, chrysophanol, emodin, physcion, polydatin, and resveratrol concentrations by 55.6, 26.5, 160.7, 622.2, 27.4, and 105.1%, respectively. Under P_0.2_ conditions, *F*. *mosseae*-inoculated plants exhibited greater chrysophanol, emodin, physcion, polydatin, and resveratrol concentrations by 42.6, 87.9, 252.3, 205.3, and 67.5%, respectively, compared with non-AM fungal plants. Aloe-emodin, emodin, physcion, polydatin, and resveratrol concentrations were significantly interacted by AM fungal inoculation and P supply (Table 1). It thus indicated that P supply and *F*. *mosseae* inoculation promoted chrysophanol, physcion, polydatin, and resveratrol concentrations of *P*. *cuspidatum*.

### Effects of AM fungi and P supply on root gene expression levels

**Figure 5:**
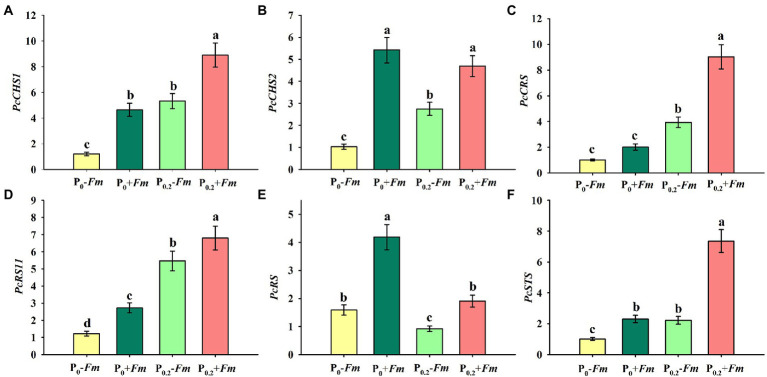
Changes in root *PcCHS1* **(A)**, *PcCHS2* **(B)**, *PcCRS1* **(C)**, *PcRS11* **(D)**, *PcRS* **(E)**, and *PcSTS* **(F)** of *Polygonum cuspidatum* by *Funneliformis mosseae* and P supply. Data (means ± SD, *n* = 3) followed by different letters above the bars indicate significant (*p* < 0.05) differences among treatments. +*Fm*, inoculation with *F. mosseae*; −*Fm*, inoculation without *F*. *mosseae*; P_0_, 0 M P; P_0.2_, 0.2 M P; P, phosphorus; CHS, chalcone synthase; RS, resveratrol synthase; STS, stilbene synthase; CRS, supposed stilbene synthase; RS11, resveratrol-forming stilbene synthase 11.

P supply induced expressions of *PcCHS1*, *PcCHS2*, *PcCRS1*, *PcRS11*, and *PcSTS* in non-AM plants, along with a decrease in *PcRS* (Figures 5A–F). Similarly, in AM plants, *PcCHS1*, *PcCRS1*, *PcRS11*, and *PcSTS* were up-regulated by P supply, coupled with no change in *PcCHS2* and inhibited expression in *PcRS*. Under P_0_ conditions, *F*. *mosseae* inoculation significantly raised root *PcCHS1*, *PcCHS2*, *PcRS11*, *PcRS*, and *PcSTS* by 2.88-, 4.26-, 1.24-, 1.64, and 0.31-fold, respectively. Under P_0.2_ conditions, *F*. *mosseae*-inoculated plants exhibited greater *PcCHS1*, *PcCHS2*, *PcCRS1*, *PcRS11*, *PcRS*, and *PcSTS* expressions by 0.67-, 0.71-, 1.30-, 0.25-, 1.08-, and 2.31-fold, respectively, compared with non-AM fungal plants. A significant interaction was observed in *PcCHS2*, *PcCRS1*, *PcRS*, and *PcSTS* expressions (Table 1). It indicated that resveratrol-associated gene expressions were involved in resveratrol accumulation.

## Discussion

Moderate P supply promoted root mycorrhizal colonization, thus promoting plant growth and root morphogenesis more prominently than single inoculation

AM fungal colonization in roots is often influenced by substrate P levels and root architecture (Wang et al., 2020). Low P levels favor root AM fungal colonization, but high P levels inhibit AM fungal colonization (Mora et al., 2008; Breuillin et al., 2010). The present study revealed that root mycorrhizal colonization rate was significantly lower at P_0_ than at P_0.2_, which is consistent with the findings of Shao et al. (2021), who reported that under P deficit conditions, P uptake by tea plants relied more on root hairs than on AM fungi, resulting in low AM fungal colonization under low P levels. Therefore, future work is needed to evaluate the response of root hairs and AM fungal colonization of *P*. *cuspidatum* to different P levels.

P supply and AM fungal inoculation collectively improved growth performance of *P*. *cuspidatum*, and the improvement was better at P_0.2_ than at P_0_. This results indicated that P is a key mineral element for vigor growth of *P*. *cuspidatum*, and P supply can promote their growth (Feng et al., 2019). On the other hand, AM symbiosis promoted the growth performance of *P*. *cuspidatum*, independent of substrate P levels, which is consistent with our earlier findings of inoculating *P*. *cuspidatum* with symbiotic fungi, including *F*. *mosseae* (Sun et al., 2022a). AM fungi also improved biomass production of *Salvia miltiorrhiza* and *Artemisia annua* plants (Huang et al., 2011; Jia et al., 2020). AM fungi can improve rhizospheric microenvironment and enhance nutrient uptake and utilization as well as water uptake by the plant after establishing a symbiosis, thus promoting plant growth (Xie et al., 2020).

Root morphology is plastic and can be influenced by various external factors (Wang et al., 2020). This study indicated that P supply distinctly promoted the establishment of root architecture, especially volume. Generally, P supply accelerates the formation and development of lateral roots to allow the root to penetrate into a larger soil volume to facilitate P acquisition (Williamson et al., 2001). Inoculation with AM fungi also dramatically improved root average diameter, total length, and projected area independent of P levels, being superior at P_0.2_ than at P_0_. Similar results were also observed in tea (Shao et al., 2021), walnut, and trifoliate orange exposed to P stress (Wu et al., 2015b; Huang et al., 2020). The improvement of root morphology triggered by AM fungi may be linked to the increase of root auxins and polyamine levels by AM fungi (Zhang et al., 2019; Zou et al., 2021).

AM fungi accelerated P acquisition of roots, associated with the release of acid and neutral phosphatase into soil

AM fungi play an essential role in nutrient acquisition by plants grown in nutrient-poor soil (Ren et al., 2022). In this study, AM fungi promoted the acquisition of root P in *P*. *cuspidatum*, accompanied by an increase in soil acid and neutral phosphatase activities, independent of substrate P levels. Phosphatases, which are P-solubilizing enzymes, are responsible for the mineralization of organic P to provide available inorganic P (Redel et al., 2019). AM soil possessed higher activities of acid and neutral phosphatase in *P*. *cuspidatum*, which can hydrolyze more organic Pi to enhance P acquisition (Hofmann et al., 2016). AM fungi secrete phosphatases and organic acids into mycorrhizosphere, and also possess phosphorus transport proteins that contribute to the uptake and transport of P, thereby enhancing P acquisition of plants (Bagyaraj et al., 2015; Yang et al., 2021).

AM fungi accelerated chrysophanol, emodin, physcion, polydatin, and resveratrol concentrations, with a higher promotion magnitude under P-deficient conditions than under adequate P conditions

In this study, HPLC was used to determine the medicinal components of *P*. *cuspidatum*, in which the concentrations of polydatin, resveratrol, and emodin were 1.89–8.64, 2.69–7.19 and 0.24–1.65 mg/g DW, respectively, which were much lower than those detected in polydatin (5.12–47.51 mg/g DW), resveratrol (3.73–15.16 mg/g DW), and emodin (2.23–17.33 mg/g DW) concentrations of *P*. *cuspidatum* by She et al. (2021) in Hubei, China. The difference could be attributed to different years of *P*. *cuspidatum* tested and different extraction methods. Future work should be made around extraction methods and gas chromatography–mass spectrometry assays. AM symbionts influence the production of secondary metabolites in various medicinal plants, and medicinal plants also regulate AM symbiosis through their secondary metabolites (Sun et al., 2021). Earlier studies also showed that *Ocimum basilicum* plants inoculated with *Glomus caledonium* had higher leaf rosmarinic and caffeic acid levels at three P levels (0.1, 0.2, and 0.3 g/kg of CaHPO_4_), as compared with uninoculated controls (Toussaint et al., 2007). *G*. *mosseae* at P levels of 40–80 mg/kg significantly promoted the accumulation of artemisinin in *Artemisia annua* plants, while high P (120 mg/kg) supply significantly reduced artemisinin content (Tan et al., 2013). In our study, P supply promoted the concentrations of aloe-emodin, physcion, and resveratrol in non-mycorrhizal plants as well as the concentrations of chrysophanol, physcion, polydatin, and resveratrol in mycorrhizal plants, implying that P is an important regulator of the synthesis of medicinal components. At P_0_ and P_0.2_ levels, AM fungi promoted chrysophanol, emodin, physcion, polydatin, and resveratrol accumulations, and the promote effect was better under P-deficient conditions than under adequate P conditions.

AM fungi up-regulated expressions of resveratrol-associated synthases, thereby promoting resveratrol production

AM fungi, after coexisting with host plants, are able to affect expressions of synthetic enzyme genes related to plant secondary metabolites (Zeng et al., 2013; Merlin et al., 2020; Oliveira et al., 2022; Sun et al., 2022a). The synthesis of resveratrol in *P*. *cuspidatum* is regulated by key enzymes such as resveratrol synthase (RS), stilbene synthase (STS), supposed stilbene synthase (CRS), and resveratrol-forming stilbene synthase (Zheng et al., 2021). In the present study, both AM fungi and P supply up-regulated *PcCRS1*, *PcRS11*, and *PcSTS* expression, except for down-regulation of *PcRS* expression, suggesting that both P supply and AM fungi were able to accelerate the expression of resveratrol-associated enzymes in *P*. *cuspidatum*, which, in turn, promoted the accumulation of resveratrol levels in roots. Although chalcone synthase (CS) is not directly involved in resveratrol biosynthesis, the enzyme can compete with RS for the same substrate (Flores-Sanchez and Verpoorte, 2009), which thus triggered down-regulated expression of *PcRS* and elevated expression of *PcCHS1* and *PcCHS2* by AM fungi and P supply. In *Paris polyphylla* var. yunnanensis plants, AM fungi also up-regulated *PpSE* expressions to accelerate polyphyllin accumulation (Li et al., 2021). Therefore, AM fungi as a biostimulator has the potential ability to regulate the expression of secondary metabolite-associated synthases, thereby promoting the level of secondary metabolites. Nevertheless, the underlying mechanisms of how AM fungi and P supply regulate the expression of these genes have yet to be studied.

## Conclusion

In short, both P supply and AM fungal inoculation were able to dramatically enhance the concentration of root medicinal components (chrysophanol, physcion, polydatin, and resveratrol) of *P*. *cuspidatum*, together with up-regulated expression of associated synthase genes. Therefore, AM fungi as a biostimulator can be introduced or appropriate P fertilizer can be provided in *P*. *cuspidatum* cultivation to promote the production of medicinal components.

## Data availability statement

The original contributions presented in the study are included in the article/Supplementary material, further inquiries can be directed to the corresponding author.

## Author contributions

R-TS and Q-SW designed the experiment. R-TS, Z-ZZ, and H-DF prepared the materials for the experiment. R-TS, M-YL, NZ, and X-CF conducted the experiment. R-TS, M-YL, and X-CF analyzed the data. R-TS wrote the manuscript. AH, EA, WH, and Q-SW revised the manuscript. All authors contributed to the article and approved the submitted version.

## Funding

This work was supported by the 2021 Undergraduate Innovation and Entrepreneurship Training Program of Yangtze University (Yz2021329). The authors extend their sincere appreciation to the Researchers Supporting Project Number (RSP-2021/134), King Saud University, Riyadh, Saudi Arabia.

## Conflict of interest

The authors declare that the research was conducted in the absence of any commercial or financial relationships that could be construed as a potential conflict of interest.

## Publisher’s note

All claims expressed in this article are solely those of the authors and do not necessarily represent those of their affiliated organizations, or those of the publisher, the editors and the reviewers. Any product that may be evaluated in this article, or claim that may be made by its manufacturer, is not guaranteed or endorsed by the publisher.

## Footnotes

^1^ www.iplant.cn
