# Supplementary material for: Arbuscular mycorrhizal fungi and phosphorus supply accelerate main medicinal component production of Polygonum cuspidatum
Source: Front Microbiol. 2022 Sep 8;13:1006140. doi: 10.3389/fmicb.2022.1006140 (PMC9493279; doi:10.3389/fmicb.2022.1006140)
Supplement: Supplementary file 1 [file Table_1.DOCX]

**Supplementary Table 1** Primer sequences of the genes used in this study for qRT-PCR

| **Genes** | **Gene ID** | **Forward sequence (5’→3’)** | **Reverse sequence (5’→3’)** |
| --- | --- | --- | --- |
| *PcCHS1* | AB019030. 1 | GTAGCTGCCGAATCTTCTACTG | TGTCGTAGCATCGTCCTTTG |
| *PcCHS2* | EU647246. 1 | GAAGCTTAAGGCGACTAGACAA | CAACCGACTTCTTCCTCATCTC |
| *PcCRS1* | DQ459350. 1 | TGAGCGAGTACGGGAATTTG | CCTTCTCCAGTCGTCTTCTTAC |
| *PcRS11* | EF117977. 1 | GATGAGATGATGAAGGCACAAAC | GGAAGTAGAAGTCGGGAAAGTC |
| *PcRS* | DQ900615. 1 | GAGATGACGAAGGCACTAACA | GGAAGTAGAAGTCGGGAAAGTC |
| *PcSTS* | EU647245. 1 | GAAGAGATGATGAAGGCACAAAC | GGAAGTAGAAGTCGGGAAAGTC |
| *PcActin* | MK288156.1 | TACAATGAGCTTCGGGTTGC | GCTCTTTGCAGTTTCCAGCT |
